# Clinician‐collected urethra swab: A good alternative sample type using cobas 4800 system for *Chlamydia trachomatis* and *Neisseria gonorrhoeae* in men

**DOI:** 10.1002/jcla.23331

**Published:** 2020-08-25

**Authors:** Qing‐Yong Wang, Lu‐Qing Zheng, Rong‐Hai Li, Ying Zheng, Xun Chen, Qi Li

**Affiliations:** ^1^ Department of Clinical Laboratory China Academy of Chinese Medical Sciences Xiyuan Hospital Beijing China

**Keywords:** *Chlamydia trachomatis*, cobas 4800 system, *Neisseria gonorrhoeae*

## Abstract

**Background:**

Nucleic acid amplification tests (NAATs) are being used increasing to detection of CT (*Chlamydia trachomatis*) and NG (*Neisseria gonorrhoeae*) infections for superior sensitivity and specificity than other tests. Male first‐void urine (FVU) sample is the optimal sample type for detection of CT and NG by NAATs. Although not being the recommended by NAATs, clinician‐collected urethra swab (CCUS) is perhaps a good alternative sample type compared with the FVU sample in men.

**Methods:**

Paired samples (FVU and CCUS) from one hundred male outpatients were simultaneously detected by urine pattern and swab pattern using cobas 4800 CT/NG assay on cobas 4800 system for the detection of CT and NG, respectively. And twenty‐one positive controls were also detected on cobas 4800 system.

**Results:**

The CT/NG cycle thresholds (Ct) value of urine pattern is lower than that of swab pattern for the same positive samples (clinical samples and positive controls) on the cobas 4800 CT/NG assay. The final CT/NG results of two sample patterns from patients were highly consistent except for four discordant results.

**Conclusion:**

CCUS is validated for a good alternative sample type for the CT/NG detection on the cobas 4800 system in this study.

## 1 INTRODUCTION

*Chlamydia trachomatis* and NG are the most prevalent bacterial sexually transmitted infections globally, about 131 million and 78 million new cases each year, respectively.^1^ In 2017, a total of 17 million CT and 555 608 cases were reported by centers for Disease Control and Prevention (CDC) in the United States, 22% and 67% increasing since 2013.^2^ If not treated early, CT/NG can cause serious health problems, painful complications, emotional injury, and so on. NAATs are being used increasing to detection of CT/NG infections for superior sensitivity and specificity than other tests. Male FVU sample is currently the optimal sample type for detection of CT/NG by NAATs.^3^ Adopted widely in numerous settings, cobas CT/NG assay for use on the cobas 4800 system provides automated solutions for the detection of CT/NG with excellent sensitivity and specificity.^4^ But in practice, the CCUS sample type should be validated by the laboratories for the male CT/NG screening using cobas 4800 system. Because clinician workers could choose the CCUS sample type for male patients in some situation (offering urine/re‐sampling difficulty, multiple items simultaneously, not likely to return for test reports, and so on). So we compared the consistent of CCUS and FVU samples using cobas CT/NG assay on cobas 4800 system (Roche Molecular Systems).

## 2 MATERIALS AND METHODS

We retrospectively the CT/NG results of one hundred paired samples (100 FVU and 100 CCUS) from male outpatients attending urology department in the hospital (mostly symptomatic) from January, 2017 to December, 2018 in this study. Samples were collected in the cobas PCR Urine Sample Kits (Roche Molecular Systems, Inc.) for the detection of CT/NG using cobas 4800 system with patients consent. Collection of FVU followed collection of CCUS in this study, all of them were transported to the laboratory at 2‐30°C for the CT/NG detection. FVU was defined as the first 10 to 50 mL of the urine stream, about 4.5 mL was added to cobas PCR Urine Sample Kit; The CCUS (provided by Jiangsu Kangjie Medical Devices Co., Ltd) transported to the laboratory was firstly placed into sterile tubes with 3 mL cobas PCR media more than 4 hours, then all of which was added to the remaining cobas PCR Urine Sample Kit before being detected. The discordant results between two sample types for male patients were judged by final clinical judgment analysis in this study. In addition, twenty‐one cobas^®^ 4800 CT/NG positive controls and positive samples were detected using cobas 4800 system by two patterns (urine and swab sample type), respectively. GraphPad Prism 8.0 software was used to plot the results. Statistical significance was set to *P* < .05.

## 3 RESULTS

The overall prevalence of CT was higher than that of NG infection (6.1% vs. 2.5%) for male patients attending the hospital during the past two years, and the median age of the positive patients was 32.7 years old. Then, the prevalence of CT (18% vs. 15%) was also higher than that of NG (9% vs. 8%) infection for CCUS and FVU of the recruited male patients in the study, respectively. And the median age of positive male patients was 33.8 years old. A total of 100 patients provided paired samples in the study, including 18 positive CCUS (15 positive FVU) for CT and 9 positive CCUS (8 positive FVU) for NG, respectively. Of them, we found four discordant results: three positive CT results from CCUS (the C_t_ value were 41.8, 40.2, and 39.8) but negative CT from FVU, and one positive NG result from CCUS (the C_t_ value was 41.2) but negative NG from FVU. The positive CT/NG CCUS from the four discordant results were right according to clinical further judgment.

**Figure 1:**
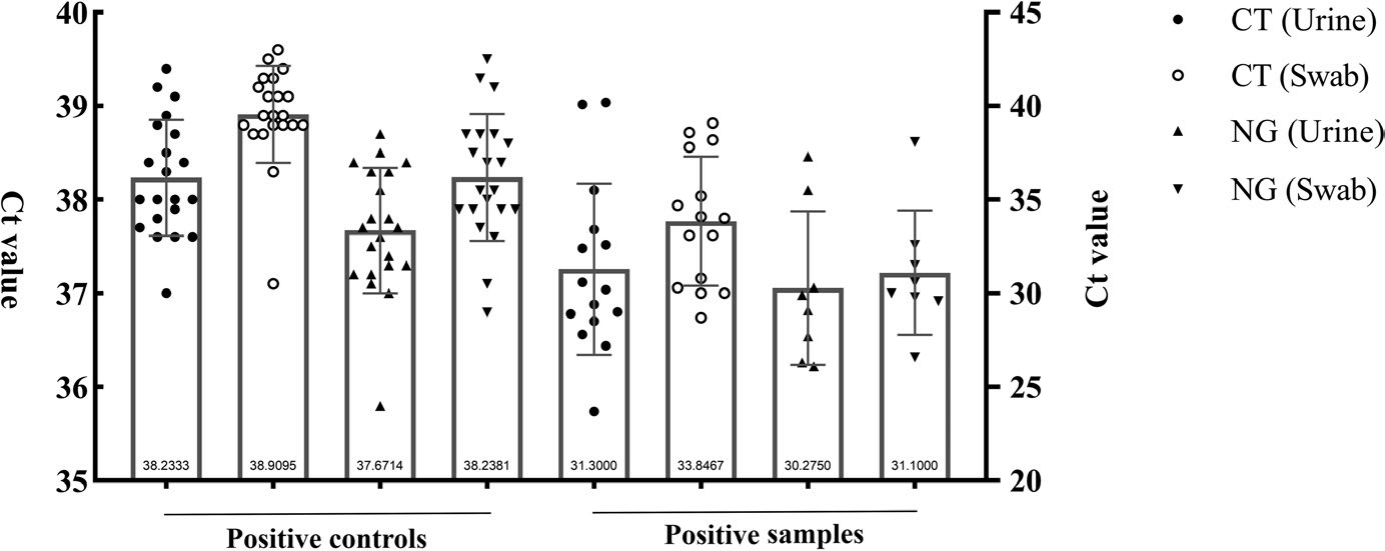
Comparison of the CT/NG C_t_ values from positive controls and positive samples by swab and urine patterns using cobas 4800 system, respectively

The sensitivity of cobas 4800 system (83.3% vs. 88.9%) for CT and NG in FVU samples showed in Table 1. The specificity and positive predictive value of CT and NG from FVU were all 100%. Then, the negative predictive value of those was 96.5% and 98.9%, respectively. Then, as shown in Figure 1, the mean C_t_ value of CT from positive controls/positive samples was 38.2 (range 37.0‐39.4 cycles)/31.3 (range 23.7‐40.2 cycles) from urine pattern was lower than 38.9 (range 37.1‐39.6 cycles) /33.8 (range 28.7‐41.8 cycles) from swab pattern, respectively (*P* < .05); and the mean C_t_ value of NG was 37.7 (range 35.8‐38.7 cycles) /30.3 (range 26.1‐37.3 cycles) from urine pattern was also lower than 38.2 (range 36.8‐39.5 cycles) /31.1 (range 26.6‐41.2 cycles) from swab pattern, respectively (*P* < .05).

**Table 1:** Performance characteristics of cobas 4800 system for clinician‐collected urethral swabs and first‐void urine samples

First‐void urine (n = 100)		Clinician‐collected urethral swab (n = 100)		Statistical data			
		Positive (n)	Negative (n)	Sensitivity (%)	Specificity (%)	PPV (%)	NPV (%)
*Chlamydia trachomatis*	Positive (n)	15	0	83.3	100	100	96.5
	Negative (n)	3	82				
*Neisseria gonorrhoeae*	Positive (n)	8	0	88.9	100	100	98.9
	Negative (n)	1	91				

Abbreviations: NPV, negative predictive value; PPV, positive predictive value.

## 4 DISCUSSION

In the United States, the rates in men infected by CT increased 39.3% than 11.1% of women during 2013‐2017, while rates of NG among gay, bisexual, and other (men who have sex with men) MSM had higher positivity rates compare to women and men who have sex with women during 2010‐2017.^2^ The date of the US CDC reports indicated that a considerable increase in male CT/NG infections in past years. FVU is currently the male optimum sample type for the routine detection of CT/NG by NATTs, accompanied by some advantages (noninvasive, self‐taken, and high organism load). But it is clear that some CT/NG infections may be missed if urine is the only sample type for the male patients, so the semen sample protocol on cobas 4800 CT/NG assay was also a good successful method for the CT detection^5^; and pharyngeal and rectal swabs had been validated by some laboratories for the detection of CT/NG in MSM.^3^, ^6^ And it is also equally importance for us to know the strengths and weakness of other alternative male sample types in addition to FVU. We believe that the CDC recommendations on the optimal male specimen type (FVU) would discourage the use of other sample types.^3^ Self‐collected specimens for the detection of CT/NG were perhaps not an effective strategy without detailed practices information.^7^ Although painful taking due to deep insertion, CCUS might provide additional information being not offered only from FVU. Interestingly, CCUS are routinely tested for bacterial or mycoplasma in most laboratories, it should be developed for detection of CT/NG on the sample type with satisfactory performance specification for NAATs. In addition, the CCUS pretreatment protocol has good practicability and conveniences.

In this study, the mean C_t_ value of CT and NG from positive controls/samples detected on urine pattern was 0.7/2.5 and 0.5/0.8 lower than those on swab pattern, respectively. C_t_ values versus DNA load are inverse, indicating that urine pattern is better than swab pattern for the same sample on cobas 4800 CT/NG assay. Obtaining one hundred paired samples is perhaps difficult for doctor workers due to expensive process and patient agreement in this study. For many years, CCUS collected from men may have posed a barrier for screening men.^8^ Cobas 4800 system offers high sensitivity, specificity, negative, and positive predictive values for the detection of CT/NG in urine specimens.^9^ And the detail preparation for CCUS in the study was not provided in the manufacturer's instructions of the cobas 4800 system. We described good concordance of CCUS with FVU on cobas 4800 CT/NG assay in this study. Four discordances of CT or NG results (CCUS samples showed C_t_ ＞ 39 cycles but the corresponding FVU samples were all negative) showed in this study. Unfortunately, we could not use more methods to confirm the real CT/NG infection of the discordance samples due to the retrospective study. But we believed that the inhibition of amplification in urine specimens for PCR and a lack of monitoring self‐collection of FVU could provide false‐negative report in some cases, and a follow‐up study for that would be performed in future. Then, the supplementary testing of NG weak‐positive samples with C_t_ ＞ 38 has limited utility^10^; and the detection of NG from rectal and urogenital samples using cobas 4800 system require no routine confirmatory testing.^11^ So being a good alternative sample type, the results from CCUS samples perhaps strengthen the identify and treatment of CT/NG infections using cobas 4800 system when applicable in the future.

One limitation of this study was the specimen size. Another limitation was not rating the collection of CCUS as easy, difficult, or neither easy nor difficult. But we believe that date presented here may be also useful for clinician workers to choose the CCUS sample type for male patients in some situation, for example, offering urine/re‐sampling difficulty, multiple items simultaneously, not likely to return for test reports, and so on.

## 5 CONCLUSION

Effective laboratory diagnosis of CT/NG infection is essential, the study showed that CCUS was a good alternative male specimen type for the detection of CT/NG using cobas 4800 system. So clinical workers could select both male sample types (FVU and CCUS) detected by NAATs if impossible.
